# Alloxan-induced diabetes exacerbates coronary atherosclerosis and calcification in Ossabaw miniature swine with metabolic syndrome

**DOI:** 10.1186/s12967-018-1431-9

**Published:** 2018-03-09

**Authors:** Jill K. Badin, Ayeeshik Kole, Benjamin Stivers, Victor Progar, Anisha Pareddy, Mouhamad Alloosh, Michael Sturek

**Affiliations:** 1 Department of Cellular & Integrative Physiology, Indiana University School of Medicine, 635 Barnhill Drive, MS 385, Indianapolis, IN 46202-5120 USA; 2 Weldon School of Biomedical Engineering, Purdue University, 206 S Martin Jischke Dr, West Lafayette, IN 47907 USA

**Keywords:** Obesity, Metabolism, Atherosclerosis, Intravascular ultrasound, Calcium dysregulation, Calcium index

## Abstract

**Background:**

There is a preponderance of evidence implicating diabetes with increased coronary artery disease (CAD) and calcification (CAC) in human patients with metabolic syndrome (MetS), but the effect of diabetes on CAD severity in animal models remains controversial. We investigated whether diabetes exacerbates CAD/CAC and intracellular free calcium ([Ca^2+^]_i_) dysregulation in the clinically relevant Ossabaw miniature swine model of MetS.

**Methods:**

Sixteen swine, eight with alloxan-induced diabetes, were fed a hypercaloric, atherogenic diet for 6 months. Alloxan-induced pancreatic beta cell damage was examined by immunohistochemical staining of insulin. The metabolic profile was confirmed by body weight, complete blood panel, intravenous glucose tolerance test (IVGTT), and meal tolerance test. CAD severity was assessed with intravascular ultrasound and histology. [Ca^2+^]_i_ handling in coronary smooth muscle (CSM) cells was assessed with fura-2 ratiometric imaging.

**Results:**

Fasting and post-prandial blood glucose, total cholesterol, and serum triglycerides were elevated in MetS-diabetic swine. This group also exhibited hypoinsulinemia during IVGTT and less pancreatic beta cell mass when compared to lean and MetS-nondiabetic swine. IVUS analysis revealed that MetS-diabetic swine had greater percent wall coverage, percent plaque burden, and calcium index when compared to lean and MetS-nondiabetic swine. Fura-2 imaging of CSM [Ca^2+^]_i_ revealed that MetS-nondiabetic swine exhibited increased sarcoplasmic reticulum Ca^2+^ store release and Ca^2+^ influx through voltage-gated Ca^2+^ channels compared to lean swine. MetS-diabetic swine exhibited impaired Ca^2+^ efflux.

**Conclusions:**

Diabetes exacerbates coronary atherosclerosis and calcification in Ossabaw miniature swine with MetS, accompanied by progression of [Ca^2+^]_i_ dysregulation in advanced CAD/CAC. These results recapitulate increased CAD in humans with diabetes and establish Ossabaw miniature swine as an animal model for future MetS/diabetes comorbidity studies.

## Background

Metabolic syndrome (MetS) affects more than one-third of all adults in the United States and is defined by the American Heart Association as the presence of three or more of the following conditions: central obesity, impaired glucose tolerance, hyperinsulinemia, dyslipidemia in the form of either elevated triglycerides or decreased HDL cholesterol, and hypertension [1]. MetS has been shown to be associated with increased risk of developing type 2 diabetes, which is rapidly increasing in incidence in the United States. Both type 2 diabetes and MetS are independently associated with increased risk of developing coronary heart disease, which continues to be the leading cause of death in the United States [1, 2].

Although studies on human subjects have consistently shown that diabetes exacerbates MetS-induced coronary artery disease (CAD) [3–7], studies in swine models have failed to reach a consensus regarding the effects of concurrent diabetes and MetS on CAD severity [8–13].

In this study, we further investigate the effects of diabetes in augmenting CAD in the Ossabaw miniature swine. The Ossabaw swine has been characterized as a clinically relevant animal model with the natural propensity to develop MetS, contributing to CAD without genetic manipulation due to their “thrifty genotype” that allows for excess fat storage [14–21]. As these swine with diet-induced MetS develop diffuse, human-like plaques [22], we tested the hypothesis that CAD will be more severe in pigs with the comorbidity of diabetes as opposed to MetS alone. We also determined whether the pattern of impaired [Ca^2+^]_i_ handling in mild through advanced CAD/CAC is noted in MetS swine with diabetes. This will further strengthen the role of Ossabaw swine as a clinically relevant animal model for investigating human CAD and studying the complex interplay between MetS and diabetes.

## Methods

### Animals and induction of diabetes

All experimental procedures involving animals were approved by the Institutional Animal Care and Use Committee at Indiana University School of Medicine with the recommendations outlined by the National Research Council and the American Veterinary Medical Association Panel on Euthanasia [23, 24]. Alloxan, a pancreatic beta cell toxin, was administered intravenously to Ossabaw miniature swine of mixed gender aged 4–7 months to induce diabetes. Briefly, alloxan (100–175 mg/kg; Sigma Chemical Co., St. Louis, MO) was dissolved in 14 mL of 1 M NaOH and 20 mL of 0.9% NaCl, for a final volume of 34 mL and a pH of 7.4. The alloxan solution was delivered through a 0.20-μm sterile filter into the jugular vein via a central venous line. To protect against possible renal toxicity, animals were given 250 mL of 0.9% NaCl through intravenous drip prior to and after alloxan delivery. The pigs were fed ad libitum and received 24 h of critical care following induction of diabetes to monitor for hypoglycemic shock. Ossabaw swine responded heterogeneously to alloxan; therefore, pigs that did not incur sufficient beta cell damage with the first alloxan dose, as indicated by normoglycemia (fasting bG < 100 mg/dL), were administered a repeat dose (75–150 mg/kg) 48 h after the initial dose. Swine were placed into two groups: non-responders that were normoglycemic (Metabolic Syndrome-Alloxan; MetS-A) and responders that were hyperglycemic, with a fasting blood glucose greater than 100 mg/dL (Metabolic Syndrome/Diabetic-Alloxan; MetS/D-A). All swine (*n* = 8 in each group) were fed a hypercaloric atherogenic diet for 6 months (1000–1350 g/day) consisting of 43% of total caloric intake from fat, 16% from protein, and 41% from carbohydrates. Swine in the MetS/D-A group received insulin glargine according to an algorithm previously published in our lab [25] to maintain glycemic control below 300 mg/dL, a clinically relevant hyperglycemic level [26]. Full insulin therapy is outlined in Table 1. Food adjustment was included to prevent wasting syndrome, a common condition seen in diabetic animals [25, 27]. For healthy control, an additional subset of Ossabaw swine (Lean; *n* = 9) were fed a standard chow diet (1000 g/day) yielding 11% of the total caloric intake from fat, 18% from protein, and 71% from carbohydrates (5L80; Purina Test Diet, Richmond, IN). A fourth group of Ossabaw swine with diet-induced MetS, without alloxan exposure, was included for metabolic comparisons (MetS; *n* = 10). Body weight in all groups was monitored weekly.

**Table 1:** Insulin therapy and feed algorithms for the maintenance of blood glucose and weight gain

Measurements			Adjustments	
Blood glucose (mg/dL)	Behavior	Body weight (kg)	Insulin glargine (U)	Food (by weight)
> Diabetic target range of 120–200	Normal		Base + 0.1 U/kg	No Change
> Diabetic target range of 120–180	Lethargic		Base + 0.2 U/kg	No Change
120–200	Normal		Base (0.1–0.3 U/kg)	No Change
< Diabetic target range of 120–200	Normal		Base (0.1–0.3 U/kg)	Increase 15%
< Diabetic target range of 120–200	Lethargic		Base (0.1–0.3 U/kg)	Increase 15%
		Decrease > 5% in one week	Base + 1 U/kg	Increase 30%
		Less than target weight gain at weeks 4, 8, 12, 16, 20	No change	Increase 15%
		Increase > 10% in 1 week	No change	Decrease 15%
		> 10% over target weight gain at weeks 4, 8, 12, 16, 20	No change	Decrease 15%

### Metabolic phenotyping

Blood was collected pre-alloxan, post-alloxan, 3 months post-diet induction, and at time of sacrifice (6 months post-diet induction) for analysis (ANTECH Diagnostics, Fishers, IN).

### Intravenous glucose tolerance test

To assess pancreatic beta cell response to glucose, a 50% glucose solution (0.5 g/kg) was injected intravenously via the central venous line. To obtain fasting glucose concentration, blood samples (3 mL) were taken at − 10, − 5, and 0 min before glucose injection, then at 5, 10, 20, 30, 40, 50, and 60 min after glucose injection. Blood glucose values were monitored by use of an Accu-Chek Advantage glucose meter, and plasma insulin values were obtained by insulin assays done at the Indiana University School of Medicine Diabetes Research Core. A tail cuff was used to measure peripheral blood pressure throughout the procedure. MetS/D-A swine did not receive their daily insulin glargine injection on the day of testing.

### Meal tolerance test

Pigs were given a standard meal (1000–1350 g chow) and allowed 45 min to eat the entire meal. Blood (3 mL) was sampled before administration of the meal (fasting) and again at 1, 2, 5, 7, and 24 h post-feeding. Blood glucose values were monitored by use of an Accu-Chek Advantage glucose meter. Eight lean swine and three swine in each the MetS, MetS-A, and MetS/D-A groups were used as a sampling group of the overall postprandial glucose clearance trend. MetS/D-A swine did not receive their daily insulin glargine injection on the day of testing.

### Intravascular ultrasound

After an overnight fast, swine were anesthetized via intramuscular injection of 2.2 mg/kg xylazine and 5.5 mg/kg Telazol (Fort Dodge Animal Health, Fort Dodge, IA). Swine were intubated and anesthesia was maintained with 2–4% isoflurane in 100% O_2_. The isoflurane level was adjusted to maintain anesthesia with stable hemodynamics. Heart rate, aortic blood pressure, respiratory rate, and electrocardiographic data were continuously monitored throughout the procedure. Following a right femoral artery cut-down, a 7 F introducer sheath was inserted for access and heparin was administered (200 U/kg). Next, a 7 F guiding catheter (Amplatz L, Cordis, Bridgewater, NJ) was advanced to the left main coronary ostium. A 3.2 F, 45 MHz intravascular ultrasound (IVUS) catheter (Revolution, Volcano, Corp., Rancho Cordova, CA) was advanced over a percutaneous transluminal coronary angioplasty guide wire and positioned in the left anterior descending (LAD) artery. Automated IVUS pullback was performed and recorded at 0.5 mm/sec and 30 frames/s. Pigs were euthanized after the IVUS procedure via cardiectomy and coronary arteries were removed for further analysis. Still frame IVUS pullback images were obtained and analyzed offline at 1 mm intervals. Percent plaque burden and calcium index measures were obtained using ImageJ software (1.48v, National Institutes of Health, USA).

### Immunohistochemistry

Sections from the tail of the pancreas were placed in 10% phosphate-buffered formalin for 24–48 h then embedded in paraffin. Tissue sections were stained with guinea pig anti-insulin ready-to-use polyclonal antibody (Agilent, Santa Clara, CA) as a marker for beta cells by the Department of Pathology at Indiana University School of Medicine (Indianapolis, IN). Images were captured using a Leica DM 3000 photomicroscope and analyzed with ImageJ software. Relative beta-cell mass was quantified by calculating the percentage of 3,3′ diaminobenzidine (DAB)-stained nuclear area to the total nuclear area using the ImmunoRatio ImageJ plugin.

### Histology

Coronary artery segments from the proximal LAD (2–4 mm in length) were placed in 10% phosphate-buffered formalin for 24–48 h, then transferred to 70% ethanol. Histology was performed in the Department of Anatomy and Cell Biology at Indiana University School of Medicine (Indianapolis, IN).

### Intracellular free calcium imaging

Whole-cell intracellular free Ca^2+^ levels were measured at room temperature (22–25 °C) by using the fluorescent Ca^2+^ indicator fura-2 AM (InCa^++^ Ca^2+^ Imaging System, Intracellular Imaging, Cincinnati, OH) as previously described [22, 28–30]. Briefly, freshly dispersed smooth muscle cells from the LAD were incubated with 3.0 μM fura-2 AM (Molecular Probes, Eugene, OR) in a shaking water bath at 37 °C for 45 min before being washed in a solution containing low Ca^2+^ concentration. An aliquot of cells loaded with fura-2 AM was placed on a coverslip contained within a constant-flow superfusion chamber that was mounted on an inverted epifluorescent microscope (model TMS-F, Nikon, Melville, NY). Cells were superfused with various solutions at a constant rate of 1–2 mL/min, including solutions that contain 80 mM K^+^ to induce Ca^2+^ influx, 5 mM caffeine to induce sarcoplasmic reticulum store release, and 2 mM barium to measure voltage-gated calcium channel activity. Fura-2 was excited by light from a 300 W xenon arc lamp that was passed through a computer-controlled filter changer containing 340 and 380 nm bandpass filters. The fluorescence emission at 510 nm was collected by using a monochrome charge-coupled device camera (COHU, San Diego, CA). Whole-cell fura-2 fluorescence was expressed as the 340 nm/380 nm ratio of fura-2 emission.

### IVUS analysis

The proximal 45 mm of the LAD was used for all IVUS analysis. All analysis was conducted by two blinded operators. To analyze for wall coverage, the circumference of the vessel cross-section was divided into 16 equal segments. Percent wall coverage was then calculated as: (total # of segments containing a thickened intimal layer ÷ 16) × 100. Wall coverage was quantified for the proximal 45 mm of the artery, in 1 mm intervals. To analyze for plaque burden, the external elastic lamina (EEL) area and lumen area were measured using ImageJ. Percent plaque burden was then calculated as: (EEL area—lumen area)/EEL area × 100. Plaque burden was quantified for the proximal 45 mm of the artery, in 1 mm intervals. To analyze for calcification, the entire IVUS pullback for the proximal 45 mm was viewed a minimum of two times. Calcification was defined as any strongly echogenic signal with acoustic shadowing. When identified, the frame numbers in which the deposit appeared and disappeared were noted to calculate the length. A representative frame of each deposit was used to calculate the arc angle using ImageJ, with each ray following the acoustic shadowing and the vertex at the center of the vessel lumen. If multiple deposits were identified per cross-section, the angles were added together. Calcium index for each artery was calculated as: (total length of calcification/45 mm) × (maximum arc angle/360°) [31, 32].

### Statistics

Statistical analysis was performed using GraphPad Prism 5.0 (San Diego, CA). Student’s t test, one-way analysis of variance (ANOVA) with Newman–Keuls post hoc analysis, or two-way ANOVA with Bonferroni post hoc analysis was performed. Data are represented as mean ± SEM. p < 0.05 was considered statistically significant.

## Results

### Ossabaw swine cardiometabolic characteristics

The swine in the MetS, MetS-A, and MetS/D-A groups were obese and had higher levels of total cholesterol compared to swine in the lean group (Table 2). The MetS/D-A group had greater total cholesterol and serum triglycerides than all other groups. The AST/ALT ratio was also increased in the MetS/D-A group as compared to the lean control, indicating possible liver dysfunction. However, there was no significant difference in creatinine and blood urea nitrogen (BUN) levels between the MetS/D-A and the lean groups, indicating normal kidney function (Table 2). Taken together, these metabolic data show that the MetS/D-A swine had more severe MetS than the other groups, despite being fed identical atherogenic diets.

**Table 2:** Metabolic characteristics of swine show hyperlipidemia and hyperglycemia in the MetS/D-A group

	Lean	MetS	MetS-A	MetS/D-A
Body weight at sacrifice (kg)	48 ± 2	87 ± 5*	73 ± 1*,^†^	70 ± 4*,^†^
Age at sacrifice (mo)	17 ± 1.4	15 ± 0.7	11 ± 0.2	14 ± 0.1
% Male	37.5	50	50	50
Creatinine (mg/dL)	0.96 ± 0.05	1.26 ± 0.13*	1.14 ± 0.07	1.06 ± 0.10
BUN (mg/dL)	14.33 ± 1.44	12.11 ± 1.65	15.00 ± 0.98	16.75 ± 0.96
AST/ALT ratio	0.95 ± 0.08	1.43 ± 0.30	1.29 ± 0.14	2.12 ± 0.49*
Serum calcium (mg/dL)	10.4 ± 0.1	10.6 ± 0.1	10.1 ± 0.1	10.3 ± 0.2
Serum phosphorous (mg/dL)	8.1 ± 0.6	7.0 ± 0.3	12.9 ± 0.7*,^†^	11.6 ± 0.7*,^†^
Total chol (mg/dL)	87 ± 3	398 ± 1*	547 ± 109*,^†^	1005 ± 158*,^†^,^‡^
Serum TG (mg/dL)	29 ± 3	42 ± 4	40 ± 6	236 ± 96*^†^,^‡^

All measurements were taken 6 months after atherogenic diet (except lean swine) and 1 week before euthanasia

Data are mean ± SEM. *MetS* metabolic syndrome, *MetS-A* metabolic syndrome-alloxan, *MetS/D-A* metabolic syndrome with diabetes-alloxan, *BUN*, blood urea nitrogen, *AST* aspartate transaminase, *ALT* alanine transaminase, *bG* blood glucose, *Chol* cholesterol, *TG* triglycerides

*p < 0.05 compared with lean swine; ^†^p < 0.05 compared with MetS swine; ^‡^p < 0.05 compared with MetS-A swine. (Lean = 9; MetS = 10; MetS-A = 8; MetS/D-A = 8.)

### Assessment of glucose clearance from a meal tolerance test

**Fig. 1:**
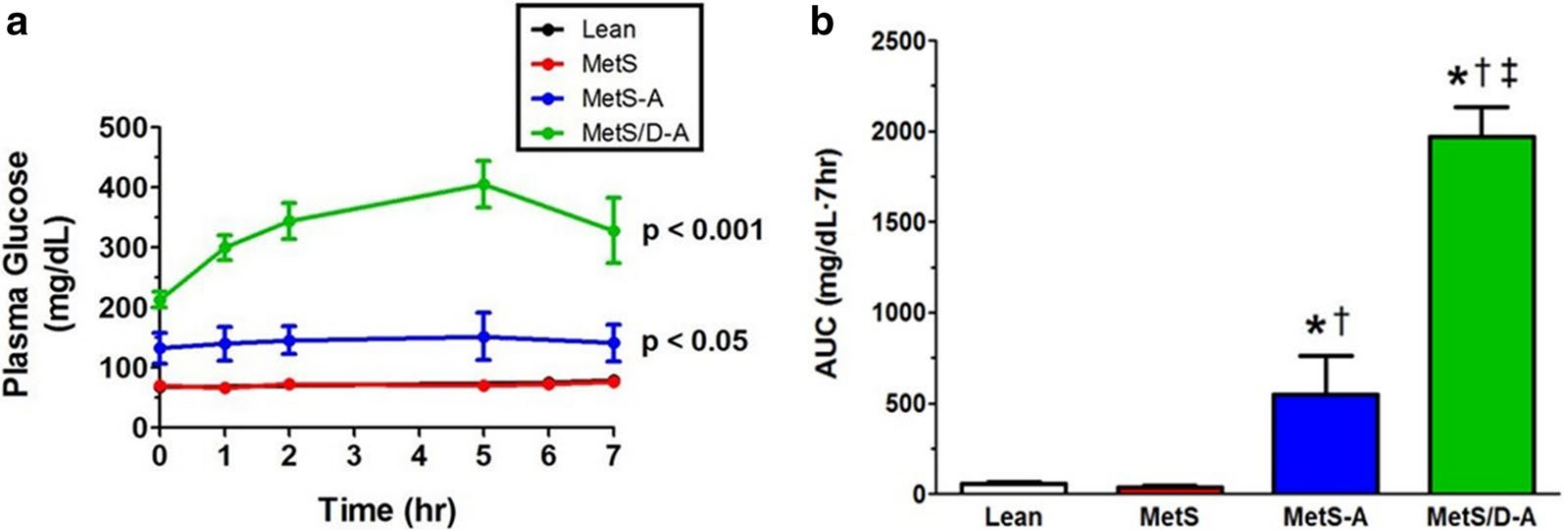
Impaired glucose clearance after a meal tolerance test in MetS/D-A swine. **a** Plasma glucose was monitored for 7 h after a meal, which revealed the lean and MetS group had comparable glucose clearance and the MetS/D-A group had impaired glucose clearance. p values are compared to lean group. **b** Area under the curve analysis shows postprandial hyperglycemia in the MetS/D-A swine as compared to all other groups. *p < 0.05 compared with lean swine; ^†^p < 0.05 compared with MetS swine; ^‡^p < 0.05 compared with MetS-A swine. (Lean = 8; MetS = 3; MetS-A = 3; MetS/D-A = 3.)

Blood glucose was monitored 7 h after administration of a meal and revealed that blood glucose measurements were significantly higher in the MetS-A and MetS/D-A swine when compared to lean swine (Fig. 1a). This is further supported by area under the curve analysis, which shows that MetS-A swine exhibit postprandial hyperglycemia as compared to the MetS and lean group, and this hyperglycemia is exacerbated in the MetS/D-A swine (Fig. 1b).

### Assessment of glucose clearance from an intravenous glucose tolerance test

**Fig. 2:**
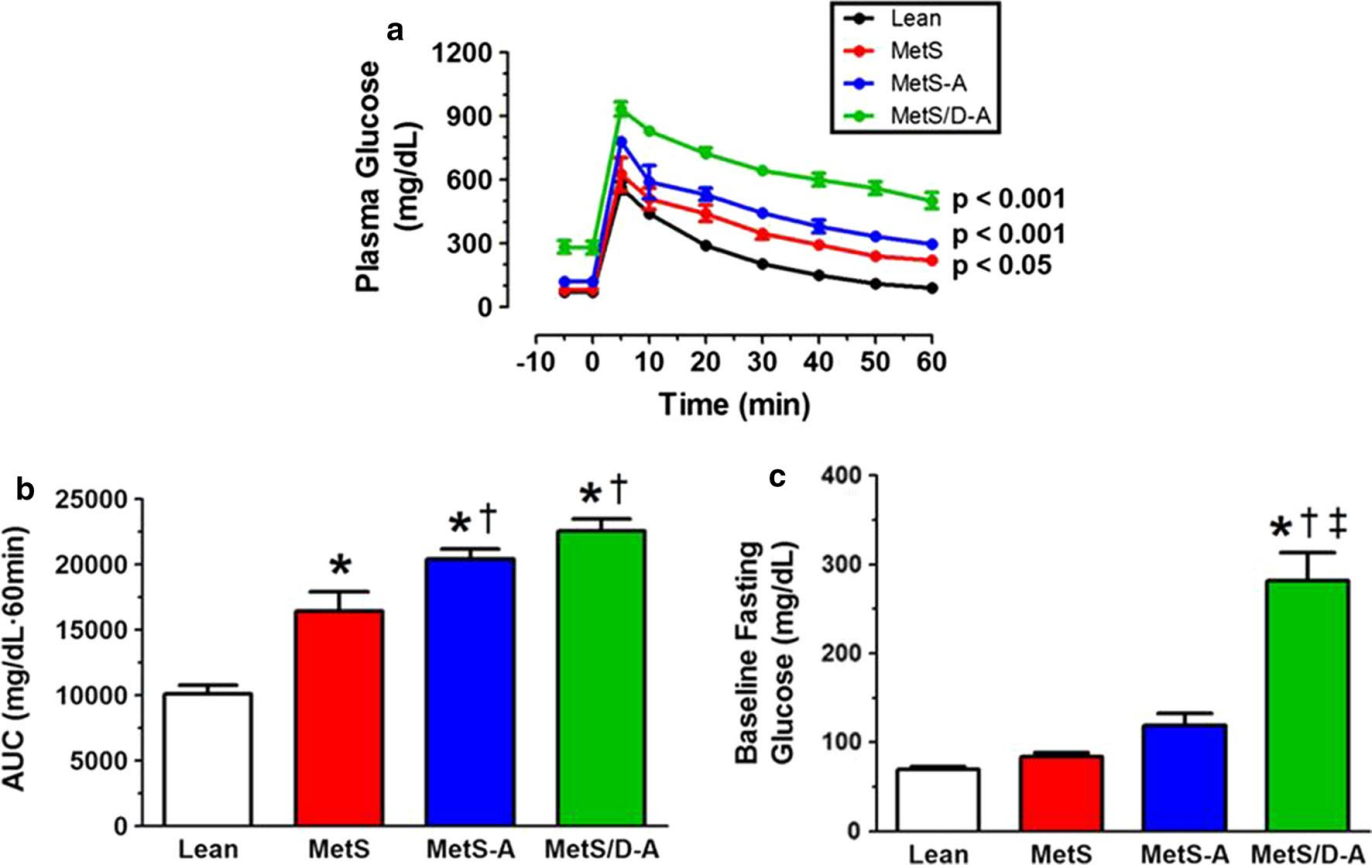
IVGTT supports the conclusion that MetS/D-A swine exhibit impaired glucose clearance. **a** Blood glucose levels were tested for 60 min after a bolus of glucose (0.5 mg/kg body weight). p values are compared to lean group. **b** Area under the curve analysis shows impaired glucose clearance in the MetS-A and MetS/D-A groups. **c** MetS/D-A swine exhibited a fourfold higher fasting blood glucose before the bolus of glucose was administered. *p < 0.05 compared with lean swine; ^†^p < 0.05 compared with MetS swine; ^‡^p < 0.05 compared with MetS-A swine. (Lean = 10; MetS = 5; MetS-A = 8; MetS/D-A = 8.)

Blood glucose was monitored 60 min after intravenous administration of a bolus of glucose and revealed that blood glucose measurements were significantly higher in the MetS, MetS-A, and MetS/D-A groups when compared to the lean group (Fig. 2a). However, while area under the curve analysis revealed that the MetS, MetS-A, and MetS/D-A groups had impaired glucose clearance when compared to the lean control group (Fig. 2b), only the MetS/D-A swine exhibited a higher fasting blood glucose over lean (Fig. 2c).

### Assessment of serum insulin levels during the intravenous glucose tolerance test

**Fig. 3:**
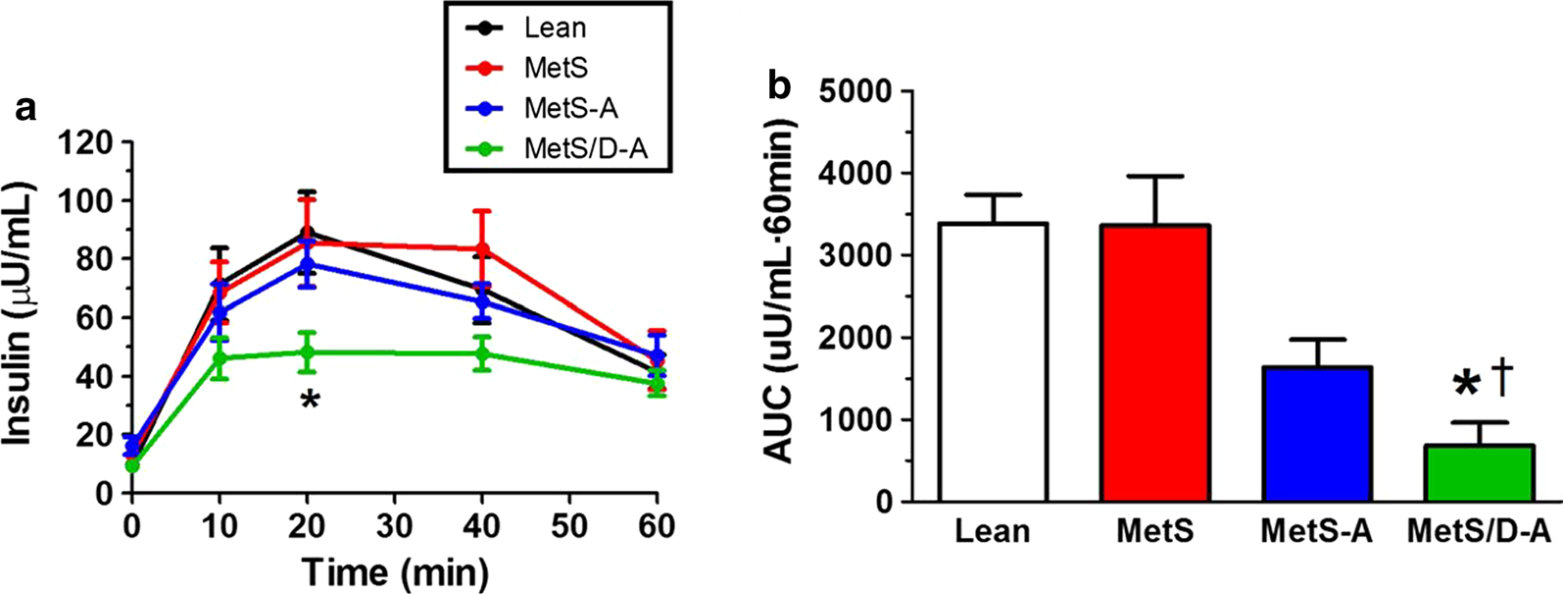
Serum insulin levels were lower in the MetS/D-A group than in the MetS-A group. **a** Insulin levels were measured at minutes 0, 10, 20, 40, and 60 during the IVGTT protocol. **b** Area under the curve analysis revealed that MetS/D-A swine exhibited hypoinsulinemia as compared to the swine in the lean and MetS groups. *p < 0.05 compared with lean swine; ^†^p < 0.05 compared with MetS swine. (Lean = 10; MetS = 5; MetS-A = 8; MetS/D-A = 8.)

Serum levels of insulin were measured during IVGTT (Fig. 3a). All swine groups except MetS/D-A showed a robust insulin peak at 20 min after the glucose bolus (Fig. 3a). Area under the curve analysis revealed that MetS/D-A swine exhibit hypoinsulinemia compared to the lean, MetS, and MetS-A groups (Fig. 3b).

### Assessment of pancreatic beta cell mass

**Fig. 4:**
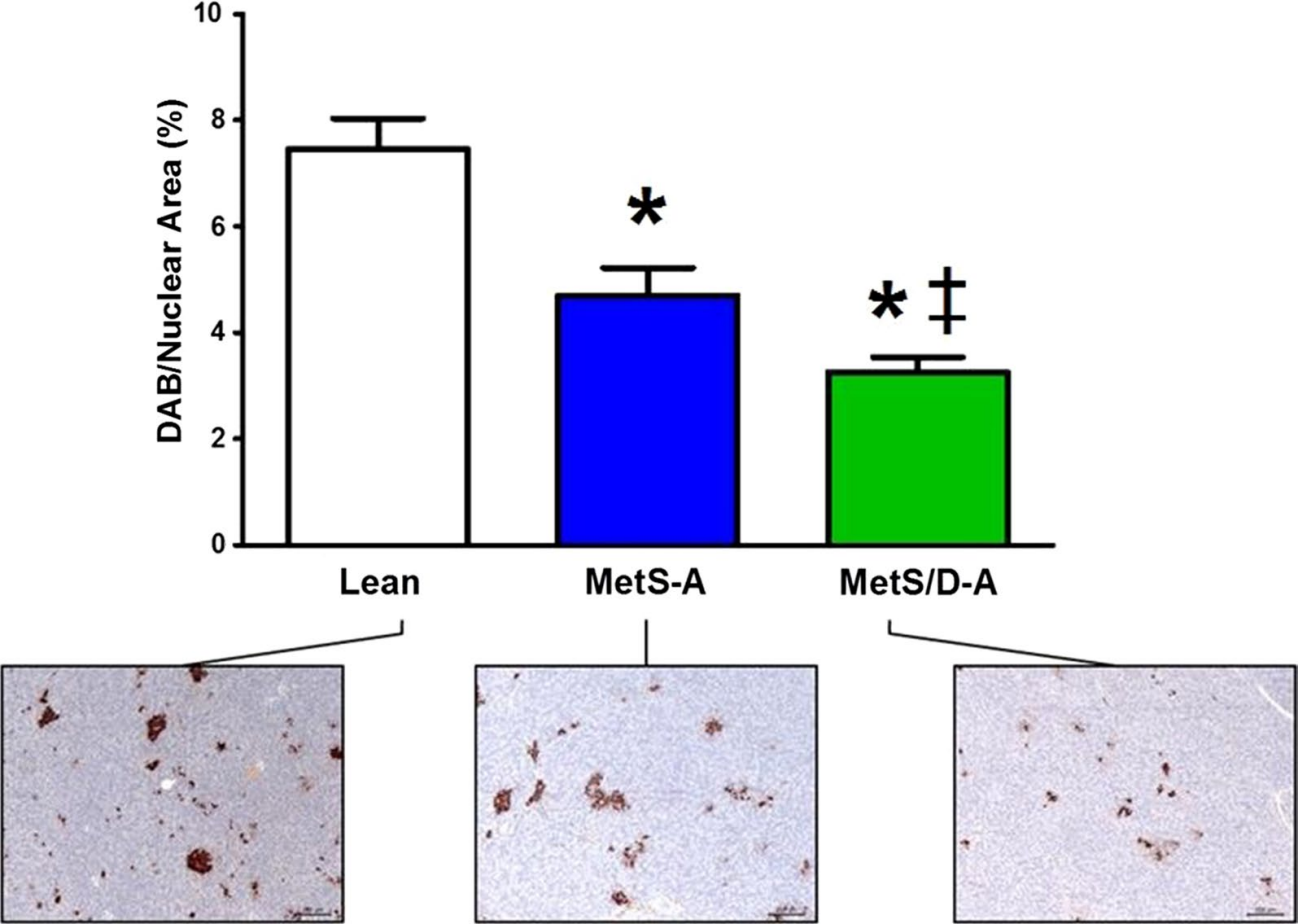
IHC shows diminished pancreatic beta cell mass in MetS-A and MetS/D-A swine. Immunohistochemical analysis using antibodies against insulin revealed that beta cell mass was decreased in MetS-A swine as compared to lean swine, and even less in MetS/D-A swine. Taken together, these data show that swine in the MetS/D-A group were indeed diabetic, due to alloxan-induced beta-cell damage. *p < 0.05 compared with lean swine; ^‡^p < 0.05 compared with MetS-A swine. (Lean = 7; MetS-A = 7; MetS/D-A = 8.)

Although the MetS-A swine did not exhibit postprandial or fasting hyperglycemia (Figs. 1b, 2c) or significant hypoinsulinemia (Fig. 3b), they show a decrease in pancreatic beta cell area compared to lean, non-alloxanized swine (Fig. 4). MetS/D-A swine show an even greater decrease in beta cell mass (Fig. 4), which is reflected in their postprandial and fasting hyperglycemia and their hypoinsulinemia (Figs. 1b, 2b, c, and 3b).

### IVUS assessment of coronary artery disease severity

**Fig. 5:**
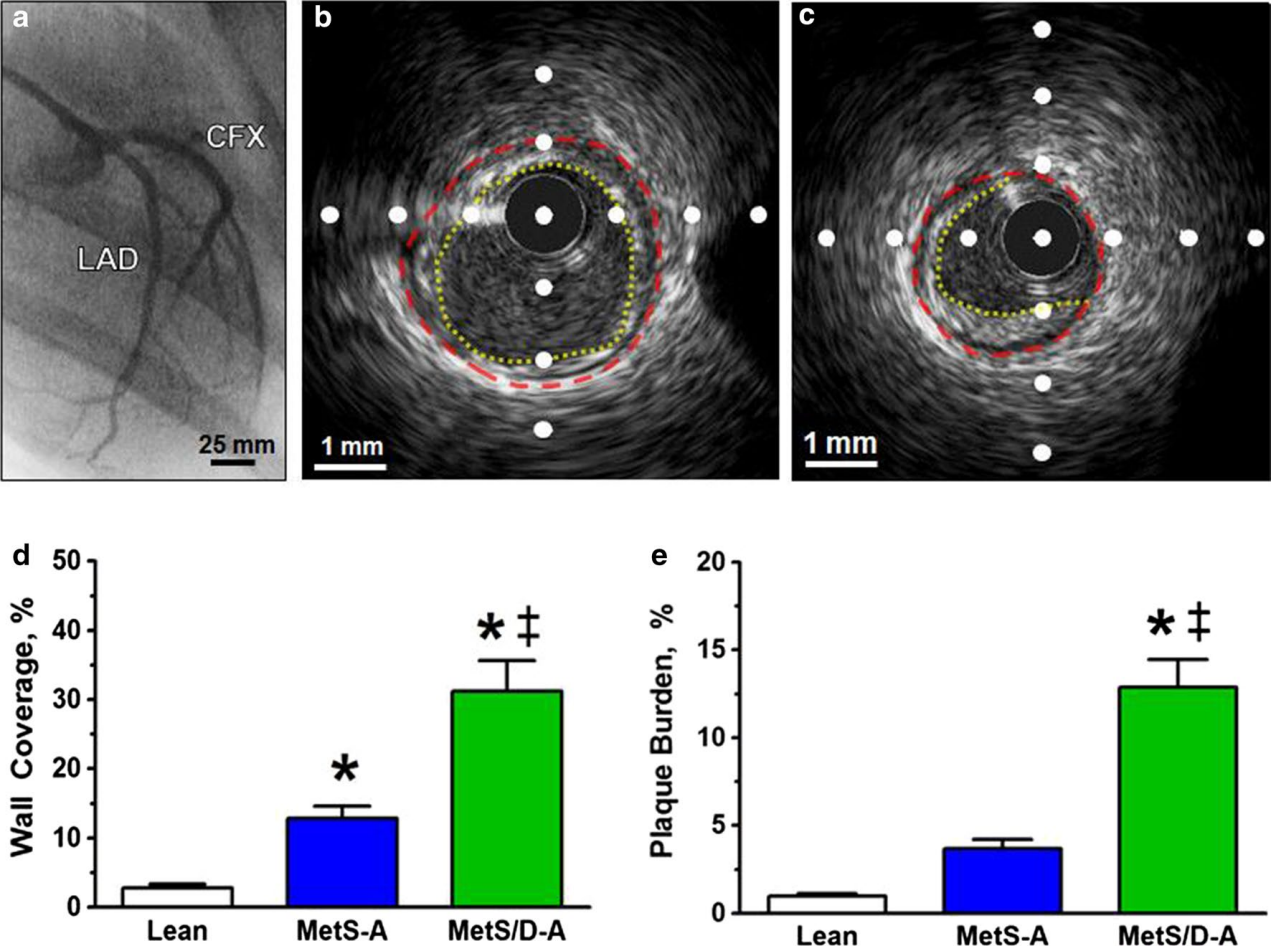
MetS/D-A swine had more advanced disease than lean and MetS-A swine. **a** Angiogram showing the LAD and CFX coronary arteries. All IVUS still frames collected for analysis were from the proximal 45 mm of the LAD. **b** Representative IVUS image of the LAD of a MetS/D-A swine showing 100% wall coverage, the percent of the circumference of the arterial wall covered by intimal thickening (original lumen highlighted in red, intimal thickening highlighted in yellow). **c** Representative IVUS image of the LAD of a MetS/D-A swine illustrating plaque burden, the percent of original lumen that is now occupied by a lesion (original lumen highlighted in red, new lumen with lesion highlighted in yellow). **d** The MetS/D-A swine had significantly greater wall coverage compared to MetS-A and lean swine. **e** The MetS/D-A swine had significantly greater plaque burden, compared to MetS-A and lean swine. *p < 0.05 compared with lean swine; ^‡^p < 0.05 compared with MetS-A swine. (Lean = 6; MetS-A = 7; MetS/D-A = 7.)

After angiography was employed to locate the LAD and circumflex (CFX) coronary arteries for catheter placement (Fig. 5a), cross-sectional images of the arteries were collected by automated IVUS pullback (representative IVUS still frames in Fig. 3b, c). MetS-A swine exhibited greater percent wall coverage compared to lean swine, while MetS/D-A swine exhibited greater percent wall coverage compared to both lean and MetS-A swine (Fig. 3d). Additionally, MetS/D-A swine exhibited greater percent plaque burden compared to lean and MetS-A swine (Fig. 3e). These data suggest that MetS/D-A swine have greater CAD severity than the lean and MetS-A groups.

### IVUS assessment of coronary artery calcification (CAC)

**Fig. 6:**
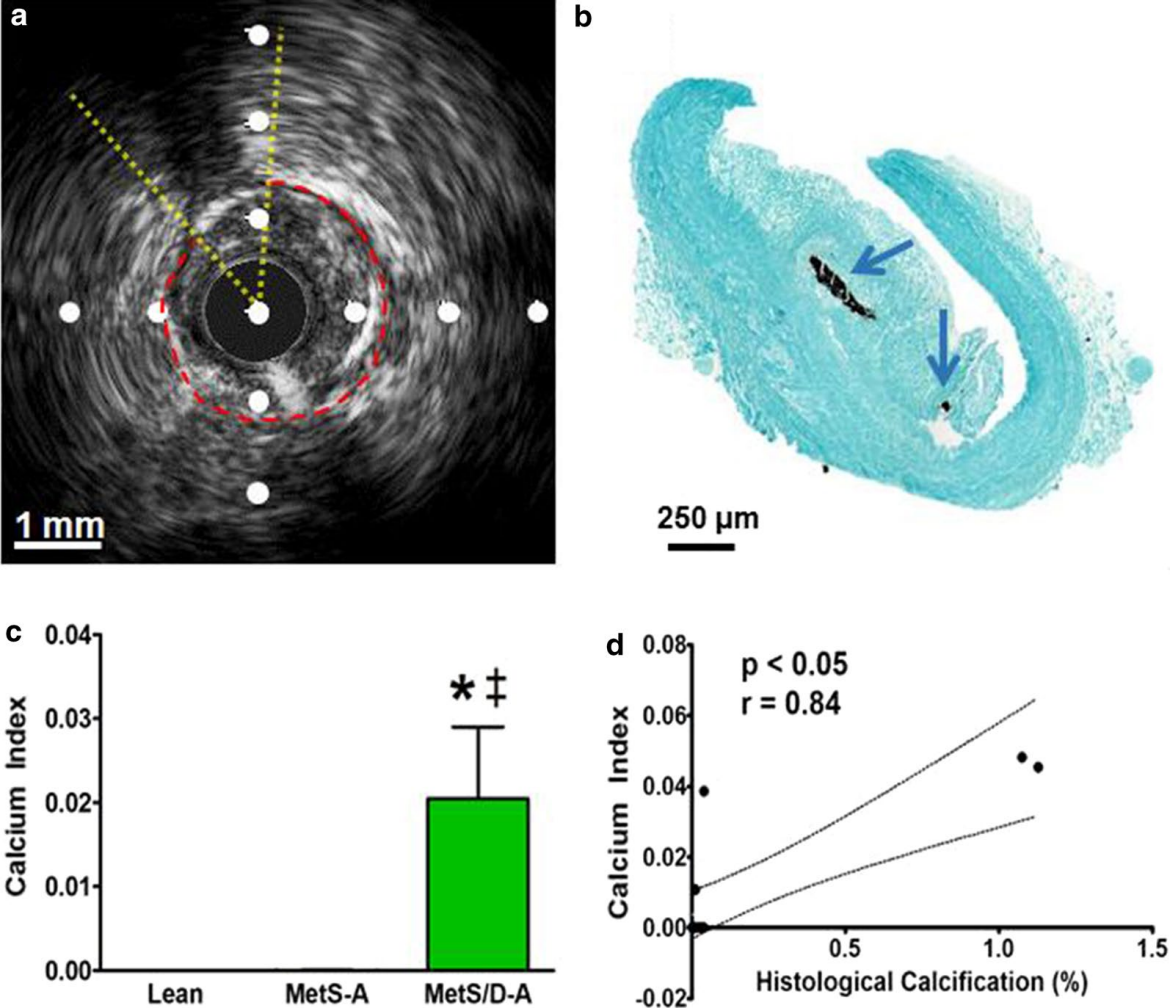
MetS/D-A swine showed greater spotty calcification compared to lean swine and MetS-A swine. **a** Representative IVUS image from the proximal LAD of a MetS/D-A swine. IVUS images were analyzed for calcification severity by using the calcium index measurement. The lumen is outlined in red, with the arc of spotty calcification and acoustic shadowing outlined in yellow. **b** Representative Von Kossa-stained histological section from the proximal LAD of a MetS/D-A swine, with noticeable spotty calcification in the neointimal layer (blue arrows). **c** Calcium index is higher in MetS/D-A swine as compared to lean and MetS-A swine. **d** Findings from analysis of IVUS data correlate to the Von Kossa histological staining analysis. *p < 0.05 compared with lean swine; ^‡^p < 0.05 compared with MetS-A swine. (Lean = 6; MetS-A = 7; MetS/D-A = 7.)

CAC was measured both by calculating the calcium index from IVUS images (Fig. 6a) and from Von Kossa histological staining (Fig. 6b). Most calcified lesions were determined to be spotty calcification. MetS/D-A swine had a significantly greater IVUS-derived calcium index than lean and MetS-A swine (Fig. 6c). The IVUS-derived measures show strong positive correlation to the percent calcification values calculated from histological analysis (Fig. 6d). These data are strong evidence that MetS/D-A swine have more severe CAC compared to the lean and MetS-A groups.

### Assessment of effects of diabetes on CSM [Ca^2+^]_i_ regulation

**Fig. 7:**
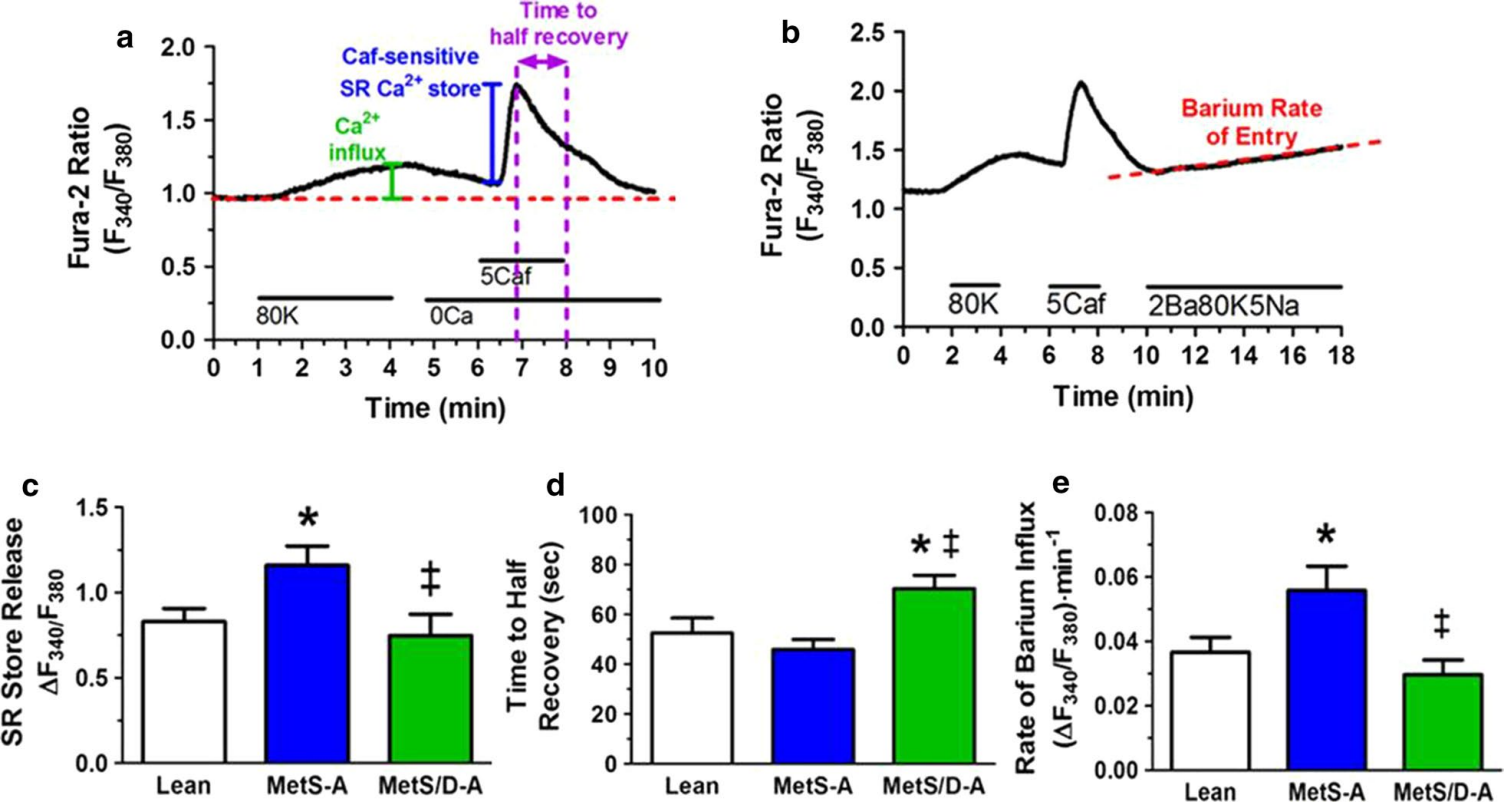
MetS-A swine showed greater SR store and VGCC function compared to lean and MetS/D-A swine. **a** After depolarizing the cell with an 80 mM K^+^ solution to induce calcium influx and maximal loading of the sarcoplasmic reticulum (SR) store, the SR store was released by activating ryanodine receptors with 5 mM caffeine. The time to half signal recovery was measured during caffeine wash-out and corresponds to the activity of calcium extrusion mechanisms. **b** A similar protocol was used with an additional exposure to a 2 mM barium solution, which enters the cell via voltage-gated calcium channels and binds to fura-2, but is not transported by Ca^2+^ ATPases. Barium as a [Ca^2+^]_i_ surrogate thereby provides a more selective measure of Ca entry. **c** The measured SR store release from CSM in MetS-A swine is greater than that seen in MetS/D-A and lean swine. This is consistent with our previous findings which show biphasic calcium handling alterations in CSM as CAD progresses in severity. **d** Extrusion mechanism activity as measured by time to half recovery is impaired in MetS/D-A swine. **e** The activity of voltage-gated calcium channels as measured by barium influx is increased in CSM from MetS-A swine and decreases back to baseline in CSM from MetS/D-A swine. This, again, is consistent with the biphasic pattern seen in CAD progression. *p < 0.05 compared with lean swine; ^‡^p < 0.05 compared with MetS-A swine. (Lean = 6; MetS-A = 8; MetS/D-A = 8.)

Figure 7a, b shows representative [Ca^2+^]_i_ responses from a CSM cell isolated from a lean swine. We assessed the caffeine-sensitive SR store release in the absence of extracellular Ca^2+^ to measure the sarcoplasmic reticulum (SR) storage capacity. The MetS-A swine had an elevated SR store that was diminished to control levels in the MetS/D-A swine (Fig. 7c). The time to half recovery to baseline was higher in the MetS/D-A swine, indicating impaired plasmalemmal [Ca^2+^]_i_ extrusion mechanisms (Fig. 7d). When voltage-gated calcium channel (VGCC) activity was assessed using a Ba^2+^ challenge, CSM cells from MetS-A swine exhibited an increased Ba^2+^ influx rate and net accumulation of Ba^2+^, but this decreased to lean control levels in the MetS/D-A swine (Fig. 7e). These data suggest that CSM [Ca^2+^]_i_ is different in animals with the comorbidities of MetS and diabetes compared to animals with only MetS.

## Discussion

There is a pressing need to establish an animal model for the common comorbidities of MetS and diabetes. This study shows for the first time that Ossabaw swine, which are a clinically relevant animal model already utilized for the study of MetS [33, 34], CAD [18, 22], CAC [17, 19], non-alcoholic steatohepatitis [16, 21, 35], and other diseases [36–38], can be used to study MetS/diabetes and the complications and mechanisms associated with those comorbidities.

Using metabolic analyses such as IVGTTs, MTTs, and insulin assays, as well as immunohistochemistry to determine pancreatic beta cell damage, we show that alloxan treatment induced a diabetic state, as defined as fasting hyperglycemia and hypoinsulinemia. Furthermore, using in vivo intravascular ultrasound imaging we show that MetS swine with alloxan-induced diabetes had increased circumferential wall coverage, plaque burden, and calcium index compared to swine with MetS alone.

While this study determined that diabetes exacerbates MetS-induced CAD in a clinically relevant porcine model, it does not delve into the mechanisms that drive this accelerated atherosclerosis. It has been reported that hyperglycemia can contribute to a dyslipidemic state by producing circulating advanced glycation end-products (AGEs) that bind lipoproteins and delay their clearance [39], generate intracellular ROS [40], and increase expression of adhesion molecules [40, 41]. Chronic high levels of glucose can also lead to mitochondrial dysfunction, resulting in increased superoxide production which, in turn, increases inflammation and ox-LDL levels [41, 42]. All these metabolic and transcriptional changes lead to the acceleration of the atherosclerotic process. Diabetes and dyslipidemia are often comorbidities due at least in part to this mechanism, and diabetic patients are at a much greater risk for developing cardiovascular diseases [41, 43].

Recently, much attention has been given to the association of perivascular adipose tissue (PVAT) and coronary atherosclerosis [44]. The causal role of PVAT in atherosclerosis was shown by surgical excision-induced attenuation of coronary atherosclerosis [45, 46]. The molecular identity of adipokines and the cross-talk between PVAT and the diseased vasculature is a growing field that has generated great interest [44]. For example, inflammation in the vasculature results in smaller, de-differentiated adipocytes around the plaque area [47]. There is evidence that PVAT potentiates leptin-induced endothelial dysfunction and increases vasomotor tone in coronary arteries of Ossabaw swine [48, 49]. Ossabaw swine can be used as a clinically relevant animal model for future studies delving into the mechanisms responsible for this bidirectional communication.

While IVUS is a robust method for determining plaque morphology in vivo, it cannot determine plaque composition with chemical specificity. This is of particular interest, as diabetes is associated with lipid laden plaques that are more vulnerable to rupture [50]. Intravascular photoacoustic ultrasound includes morphological imaging and has chemical specificity to determine lipid content inside atherosclerotic plaque [51–54]. This advancement will enable longitudinal characterization of plaque composition in vivo during progression of coronary atherosclerosis and calcification [51].

Ca^2+^ is an important second messenger that plays a vital role in contraction [55, 56], proliferation [55, 57], migration [58, 59], and transcription [60, 61]. Recently, our lab has clarified that CSM [Ca^2+^]_i_ handling dysfunction occurs in a biphasic manner during CAD progression, with SR Ca^2+^ store capacity and sarco-endoplasmic reticulum Ca^2+^ ATPase (SERCA) function being upregulated in early, mild CAD and downregulated in late, more severe CAD [18]. Additionally, it has previously been shown that plasmalemmal Ca^2+^ extrusion mechanism function, as measured by the time to half recovery, is decreased in advanced disease [28]. These Ca^2+^ handling alterations can be seen in CSM from MetS-A swine, which exhibited changes associated with mild CAD, and in CSM from MetS/D-A swine, which exhibited changes associated with more severe CAD.

Increased VGCC and SERCA function are associated with greater CSM proliferation [18]. While the MetS-A swine had increased VGCC and SERCA function, they only exhibited greater percent wall coverage compared to lean swine. MetS/D-A swine exhibited both greater percent wall coverage and percent plaque burden, even though their VGCC and SERCA activity was comparable to lean swine. This could be due to the severity of the metabolic conditions, which is proportional to the duration of MetS. The MetS/D-A swine could have exhibited a longer period of CSM proliferation before VGCC and SERCA activity decreased back down to baseline, while the MetS-A swine were still undergoing proliferation at the time of euthanasia. Future studies should investigate the effect of the diabetic state on CSM proliferation.

High serum Ca^2+^ and phosphorous may contribute to the increased CAC seen in the MetS/D-A swine [62]. However, serum Ca^2+^ is not significantly elevated when compared to the lean swine and, while serum phosphorous is elevated in the MetS/D-A swine, it is still comparable to the MetS-A group. Therefore, the greater CAC seen in the MetS/D-A group cannot be contributed to the uremic milieu alone. It is hypothesized in several papers that impairments in [Ca^2+^]_i_ buffering can lead to Ca^2+^ overload and subsequent vascular calcification [62, 63]. The changes in SERCA and VGCC function and the dysfunction seen in the calcium extrusion mechanisms, which include the sodium-calcium exchanger and the plasma membrane calcium ATPase, could lead to the increased calcification seen in histology and IVUS analysis. However, even though MetS/D-A swine have impaired Ca^2+^ extrusion mechanisms, they only exhibit spotty calcification. Calcification is “spotty” if the arc of calcium is less than 90° [32]. Spotty calcification has been reported to destabilize atherosclerotic plaques and increase the incidence of acute myocardial ischemia [64, 65]. Therefore, although spotty calcification is a precursor to macrocalcification, it has serious clinical implications [66]. Future studies should focus on [Ca^2+^]_i_ dysregulation and vascular calcification in diabetic swine fed an atherogenic diet for a longer period of time.

Overall, we found that MetS swine with alloxan-induced diabetes had greater CAD severity and calcium handling that was indicative of severe CAD while normoglycemic MetS swine showed less severe CAD and calcium handling that was indicative of mild CAD. This mirrors the patterns seen in human studies [3–7] and establishes Ossabaw swine as a relevant animal model for MetS/diabetes.

There is no shortage of literature investigating the role of diabetes in exacerbating CAD in the context of MetS in swine models. However, there have been conflicting results in these studies. Gerrity et al. showed that diabetic/hyperlipidemic Yorkshire pigs developed more stenotic and advanced atherosclerotic lesions, compared to the nondiabetic/hyperlipidemic control group [8]. Additionally, our group has shown that the induction of diabetes in Sinclair and Yucatan miniature pigs with hyperlipidemia leads to increased CAD development [10, 11]. In contrast, a recent study by Al-Mashhadi et al. showed that Yucatan minipigs with a PCSK9 gain-of-function mutation developed severe hyperlipidemia, but no augmentation of CAD with the induction of diabetes by streptozotocin (STZ) [9]. However, because of the dramatic effect increase in LDL cholesterol levels and subsequent CAD development in this transgenic model, it is unclear if a contribution of hyperglycemia to atherogenesis was present, but masked by the severity of disease. Ludvigsen et al. showed that diet-induced atherosclerosis in Gottingen minipigs was not augmented by STZ-induced diabetes, but the sample size in this study (*n* = 6) was not large enough to be conclusive [12]. Interestingly, another study from our group found that in Yucatan swine diabetes without dyslipidemia was not enough to increase early atheroma [13]. This study also showed that hyperlipidemic diabetic swine did not exhibit greater CAD severity than swine with hyperlipidemia alone [13]. This may have been because of the large effect of plasma lipids that nullified the synergistic effects of diabetes.

None of the previously mentioned studies used Ossabaw miniature swine, for which there is remarkable similarity to human MetS and CAD [22]. Ossabaw swine develop more severe CAD with diabetes on a MetS background, which is similar to results seen in human patients. Thus, this study provides support for Ossabaw swine as an excellent model for translation to human clinical medicine. Taken together, the data in this study provide evidence supporting the use of Ossabaw swine in future studies that investigate mechanisms or outcomes of diabetes superimposed on a MetS background. By having a reliable, clinically relevant animal model that recapitulates human disease we can be far more certain of the translatability of our research.

## Conclusion

In conclusion, swine exhibiting both MetS and alloxan-induced diabetes have more severe CAD and coronary artery calcification when compared to swine with only MetS as measured by IVUS. Additionally, coronary smooth muscle from swine with MetS alone show similar calcium handling alterations as mild CAD, and swine with both MetS and diabetes show similar calcium handling alterations as more severe CAD. Ossabaw swine, similar to humans, reliably develop more severe CAD with the comorbidities of diabetes and MetS, which supports their use as a clinically relevant animal model for future studies investigating the mechanisms of diabetes superimposed on a MetS background.

## Abbreviations

ALT: alanine transaminase
AST: aspartate transaminase
bG: blood glucose
BUN: blood urea nitrogen
[Ca^2+^]_i_: intracellular free calcium
CAD: coronary artery disease
CAC: coronary artery calcification
Cfx: circumflex artery
Chol: cholesterol
CSM: coronary smooth muscle
DAB: 3,3′ diaminobenzidine
EEL: external elastic lamina
IHC: immunohistochemistry
IVGTT: intravenous glucose tolerance test
IVUS: intravascular ultrasound
LAD: left anterior descending artery
MetS: metabolic syndrome
MetS-A: metabolic syndrome-alloxan
MetS/D-A: metabolic syndrome/diabetes-alloxan
MTT: meal tolerance test
SERCA: sarco-endoplasmic reticulum Ca^2+^ ATPase
SR: sarcoplasmic reticulum
STZ: streptozotocin
TG: triglycerides
VGCC: voltage-gated calcium channel
